# PanFP: pangenome-based functional profiles for microbial communities

**DOI:** 10.1186/s13104-015-1462-8

**Published:** 2015-09-26

**Authors:** Se-Ran Jun, Michael S. Robeson, Loren J. Hauser, Christopher W. Schadt, Andrey A. Gorin

**Affiliations:** Comparative Genomics Group, Bioscience Division, Oak Ridge National Laboratory, Oak Ridge, TN USA; Joint Institute for Computational Sciences, University of Tennessee, Knoxville, TN USA; Systems Genetics, Biosciences Division, Oak Ridge National Laboratory, Oak Ridge, TN USA; Fish, Wildlife and Conservation Biology, Colorado State University, Fort Collins, CO USA; Department of Microbiology, University of Tennessee, Knoxville, TN USA; Computer Science and Mathematics Division, Oak Ridge National Laboratory, Oak Ridge, TN USA

**Keywords:** Microbial communities, Metagenome, 16S rRNA survey, Pangenome

## Abstract

**Background:**

For decades there has been increasing interest in understanding the relationships between microbial communities and ecosystem functions. Current DNA sequencing technologies allows for the exploration of microbial communities in two principle ways: targeted rRNA gene surveys and shotgun metagenomics. For large study designs, it is often still prohibitively expensive to sequence metagenomes at both the breadth and depth necessary to statistically capture the true functional diversity of a community. Although rRNA gene surveys provide no direct evidence of function, they do provide a reasonable estimation of microbial diversity, while being a very cost-effective way to screen samples of interest for later shotgun metagenomic analyses. However, there is a great deal of 16S rRNA gene survey data currently available from diverse environments, and thus a need for tools to infer functional composition of environmental samples based on 16S rRNA gene survey data.

**Results:**

We present a computational method called pangenome-based functional profiles (PanFP), which infers functional profiles of microbial communities from 16S rRNA gene survey data for Bacteria and Archaea. PanFP is based on pangenome reconstruction of a 16S rRNA gene operational taxonomic unit (OTU) from known genes and genomes pooled from the OTU’s taxonomic lineage. From this lineage, we derive an OTU functional profile by weighting a pangenome’s functional profile with the OTUs abundance observed in a given sample. We validated our method by comparing PanFP to the functional profiles obtained from the direct shotgun metagenomic measurement of 65 diverse communities via Spearman correlation coefficients. These correlations improved with increasing sequencing depth, within the range of 0.8–0.9 for the most deeply sequenced Human Microbiome Project mock community samples. PanFP is very similar in performance to another recently released tool, PICRUSt, for almost all of survey data analysed here. But, our method is unique in that any OTU building method can be used, as opposed to being limited to closed-reference OTU picking strategies against specific reference sequence databases.

**Conclusions:**

We developed an automated computational method, which derives an inferred functional profile based on the 16S rRNA gene surveys of microbial communities. The inferred functional profile provides a cost effective way to study complex ecosystems through predicted comparative functional metagenomes and metadata analysis. All PanFP source code and additional documentation are freely available online at GitHub (https://github.com/srjun/PanFP).

**Electronic supplementary material:**

The online version of this article (doi:10.1186/s13104-015-1462-8) contains supplementary material, which is available to authorized users.

## Background

The complexity of microbial communities has been well studied for decades, which reveals relationship between changes in microbial communities and ecosystem functions. Using DNA sequencing technologies, microbial communities can be explored by sequencing the amplified fragments of phylogenetic marker genes (for example, 16S rRNA gene data) or all detectable DNA fragments extracted from the environmental samples (called metagenomes or shotgun metagenomes) [1, 2]. The 16S rRNA gene community data, a rapid and cost-effective approach, is commonly used to assess bacterial abundance and diversity in communities because 16S RNA occurs universally and is highly conserved among all species of Bacteria and Archaea [3]. Using existing technologies, these statistically robust data can be obtained for community studies over large numbers of samples, and thousands of 16S rRNA genes are obtained for each sample. However, the functional capabilities of these communities, for example the metabolic potential for nitrogen fixation, cannot be easily derived from 16S rRNA gene studies [4]. Metagenomics provides a direct view of the communities’ gene content and thus their functional capability. Such studies aim to understand how microbes interact, and perform complex functions in a variety of environments through answering questions such as, what species are present and abundant, as well as what functions are present or absent based on identification of a panel of microbial organisms, genes, variants, pathways, or metabolic functions [5, 6]. Although the generation of metagenomic data has significantly increased as a result of on-going development of high-throughput sequencing technologies, obtaining enough sequencing data to describe the metagenome of a highly diverse sample, *en toto*, is still cost prohibitive. Cost, computational time, and statistical robustness becomes even more intractable for large-scale studies [7]. However, several recent studies have indicated that the functional content of complex communities remains more constant than the phylogenetic composition of the community [8, 9].

Given the above, there is a need for the development of methods to better bridge the gap between 16S rRNA gene and metagenomics methods. Here we present a method for inferring the potential functional compliment of a microbial community based on the phylogenetic marker gene 16S rRNA. These predictive tools are a cost effective way to expand the utility of 16S rRNA gene-based studies and allow the development of functional hypotheses for such communities using automated methods.

## Method

Here, we present a new computational method called pangenome-based functional profile (PanFP), which infers the functional profiles of microbial communities based on 16S rRNA gene survey data. Our method takes the measured abundance profile of detected operational taxonomic units (OTUs), and produces a functional profile of controlled vocabulary terms with the expected abundance for the studied community. The taxonomic groupings of organisms are commonly recognized as the reflection of evolutionary relationship of organisms encoded by the shared functional content [10]. Our approach ultimately relies on the taxonomic lineages of OTUs. To reflect the underlying functional capabilities of microbial communities through the standard representation of genes and gene product attributes, we utilize KEGG Orthology database [11] of controlled vocabulary of functional terms (called KO terms) for all predicted proteins and functional RNAs. However, our approach naturally extends to other gene annotation databases including Gene Ontology [12], Pfam [13], TIGRFAMs [14] in a like manner. We validated PanFP in comparison with sequenced metagenomes and an existing method, PICRUSt [4] using 65 different environmental and mock community samples derived as part of the Human Metagenome Project (HMP) and other projects.

## Algorithm details

### A flow diagram for PanFP is depicted in Additional file 1: Figure S1

#### Prerequisite: prokaryote genomes with functional annotation

PanFP is not limited by the specific completion of a set of reference genomes, as taxonomically related genomes are merged into a pangenome in a straightforward way. This allows for the integration of all currently available functionally annotated genomes (complete and incomplete) into our analysis pipeline. For the purposes of this study, we only considered complete genomes of prokaryotes with their full taxonomic lineages [downloaded from the National Center for Biotechnology Information (NCBI) through ftp://ftp.ncbi.nih.gov/genomes/Bacteria on Oct, 2013]. We also considered only chromosomally encoded sequences due to the dominant role of plasmids in horizontal gene transfer [15]. If an organism had multiple chromosomes, a single merged genome was assigned to the organism. We mapped KO terms to proteins using the cross-reference ID mapping between NCBI Refseq and UniProt provided by UniProt KnowledgeBase (UniProtKB) database [16]. The proteins can have multiple functional roles, and thus be annotated with multiple functional terms. However, about 33 % of genes in Bacterial and Archaeal complete genomes in NCBI are annotated as “hypothetical”, and many genes are annotated with very little information, which results in many proteins without assigned functional terms [17]. The functional coverage is defined as the number of proteins with functional annotations divided by the total number of proteins for a complete genome. The distribution of functional coverage of organisms is shown in Additional file 1: Figure S2. Currently in our approach, only prokaryote genomes with at least 30 % of functional coverage are accepted in the pipeline to avoid the underestimation of frequency of functional genes by poor annotations of genomes pooled for pangenome construction, which resulted in ~2400 organisms. With a 10 % coverage cutoff, we retained one more complete prokaryotes compared to a 30 % cutoff that we employed almost all annotated genomes of complete prokaryotes. The 16S rRNA gene survey data (also called the OTU-sample table or OTU table) can be made by clustering 16S rRNA gene sequences at a user-defined similarity threshold through a variety of OTU picking strategies that are available via tools like QIIME [18]. QIIME provides three high-level protocols for OTU picking: de novo, closed-reference, and open-reference. The closed-reference OTU picking protocol only retains OTUs that have a positive hit against a set of reference sequences (e.g. Greengenes) within a provided similarity threshold. Note that PanFP is not limited to a specific OTU-picking strategy since our method is based on the pangenome construction through OTUs’ taxonomic lineages.

##### STEP 1: trim OTU’s taxonomic lineage

At this step, we performed two tasks to fully employ our database of complete prokaryotes with functional annotation and taxonomy, which were extracted from NCBI. First, when discrepancies between OTUs’ taxonomic lineages generated by the user’s OTU processing pipeline and NCBI taxonomy are observed, the OTUs’ lineages are corrected as follows: For example, an OTU belongs to a genus group recognized by NCBI taxonomy, but to a family group that is not recognized on NCBI taxonomic hierarchy probably due to the taxonomic nomenclature updates. Then we modify the OTU’s taxonomic lineage to that above the genus level according to NCBI taxonomy. Second, we trim OTUs’ taxonomic lineages from the lowest level until at least one prokaryote genome belonging to the taxon is identified. For example, assume that a given OTU has the followinglineage: kingdom:Bacteria; phylum:Firmicutes; class:Bacilli; order:Bacillales; family:Planococcaceae; genus:*Kurthia*. Then, we trim the OTU’s lineage by abandoning a rank at the genus level since there are no organisms of this genus in our database of prokaryotic genomes. The OTU’s newly trimmed taxonomic information contributes to the inference of functional profiles. Thus, our method is not limited to a set of specifically completed prokaryote genomes, which allows the use of all available complete or incomplete genomes. Finally, we keep OTUs whose lineages contain at least a phylum level designation. The HMP mock community used in the study was composed of 158 OTUs with lineages up to species, 412 OTUs with lineages up to genus, 67 OTUs with lineages up to family, 12 OTUs with lineages up to order, one OTU with lineage up to class, two OTUs with no taxonomic information. For this data, after modifying OTUs’ lineages in STEP 1, we ended up 68 OTUs with lineages up to species, 452 OTUs with lineages up to genus, 114 OTUs with lineages up to family, 15 OTUs with lineages up to order, one OTU with lineage up to class, indicating that information loss of the user’s chosen taxonomy against the NCBI taxonomy mostly occurred at the species level.

##### STEP 2: make a lineage-function table

For each OTU, PanFP builds a pangenome by making a superset of all genes present in organisms pooled from the dataset of prokaryote genomes at the given OTU’s taxonomic lineage. Therefore, the OTUs with the same taxonomic lineages have the same pangenome. Then, PanFP derives a functional profile of the pangenome by accumulating functional compositions in the superset. In such a case that horizontal gene transfer events occur only within the OTU’s taxon that genes in genomes pooled at the OTU’s taxon are transferred to other genomes pooled at the OTU’s taxon, and no genes from genomes outside the OTU’s taxon are transferred to genomes pooled at the OTU’s taxon, our method is not affected by horizontal gene transfer since genes horizontally transferred within the OTU’s taxon are still found in a pangenome for the OTU. The functions shared by more organisms in the superset have higher occurrence. We assign the pangenome’s functional profile normalized by the number of pooled organisms to the OTU’s lineage:

$$fq(lineage_{i} ,KO_{j} ) = \frac{1}{{O_{i} }}occurrence(KO_{j} \;in\;pangenome_{i} )$$

where *pangenome*_*i*_ is the pangenome for the *lineage*_*i*_, and *O*_*i*_ is the number of organisms pooled at the *lineage*_*i*_. The number of organisms available for the OTUs’ taxonomic lineage, *O*_*i*_, could vary dramatically across different lineages.

##### STEP 3: normalize OTU’s abundance

The OTU abundance reflects 16S rRNA full number of occurrences (e.g. sequence count or relative abundance) of organisms assigned to a given OTU. To implement organismal abundance, we divide the OTUs’ abundance by the putative 16S rRNA gene copy number, which is defined as the median of 16S rRNAs copy number of the organisms pooled at the OTU’s taxonomic lineage. Again, all OTU frequencies for a given sample are normalized by the sample size such that the resulting frequencies, *fq* (*OTU*_*i*_, *sample*_*j*_) could be directly comparable between different samples.

##### STEP 4: convert an OTU-sample table into a lineage-sample table

An OTU-sample table is converted into a lineage-sample table by summing the frequencies of OTUs with the same lineage in a sample, as follows:

$$fq(lineage_{i} ,sample_{j} ) = \sum\limits_{{OTU_{k} \;has\;lineage}} {fq(OTU_{k} ,sample_{j} )}$$

##### STEP 5: convert a lineage-sample table into a function-sample table

We derive a function-sample table by combining functional profiles of lineages with weights corresponding to the lineage abundance in the sample:

$$fq(KO_{i} ,sample_{j} ) = \sum\limits_{{lineage_{k} }} {fq(lineage_{k} ,sample_{j} ) \times fq(lineage_{k} ,KO_{i} )}$$

##### STEP 6: assessing the uncertainty in function-sample estimates

To assess the uncertainty in function-sample estimates under the assumption that each OTU abundance profile contributes to functional terms’ profile independently, we performed bootstrapping that we selected OTUs along with their abundance across samples (i.e., rows in the OTU-sample table) as many as the number of OTUs in the OTU-sample table (i.e., the number of rows in the OTU-sample table) randomly with replacement. Therefore, some OTUs in the OTU-sample table can be represented more than once and some not at all. We produce 100 bootstrap replicates of the OTU-sample table, and corresponding 100 function-sample tables through STEP 1–STEP 5. We examine whether the estimates of functional terms neither belong to the top 2.5 % nor the bottom 2.5 % (i.e., at a 95 % confidence interval). For all of test sets used in the study (see “Case study” section), all functional terms identified in STEP 5 had estimates within the 95 % confidence interval. Note that a software, PanFP does not include a step for assessing the uncertainty in function-sample estimates by bootstrapping.

## Case study

**Fig. 1:**
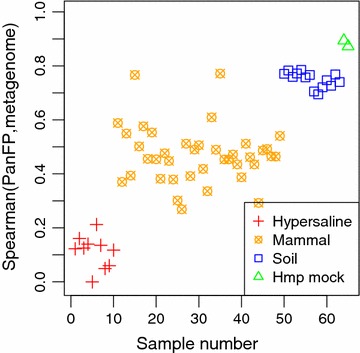
Spearman correlation between functional profiles inferred by PanFP and sequenced metagenomic functional profiles for 65 samples

We applied PanFP to diverse communities, which include 39 mammal gut samples, 14 soil samples, ten hypersaline microbial mats, and two synthetic mock communities from the HMP where both 16S rRNA gene data and metagenome data have been derived from the same samples. This is the same dataset used in a recently developed method, PICRUSt [4, 19]. To evaluate our method, we compared functional profiles inferred by PanFP to the sequenced metagenomes for 65 samples where PICRUSt annotated whole genome sequencing reads to KO terms using HUMAnN [20]. The number of KO terms present in the 65 sequenced metagenomes is described in Additional file 1: Table S1. The hypersaline metagenomes have the range of 263 ± 131 different KO terms, mammal gut metagenome of 1190 ± 640 KO terms, soil metagenomes of 4328 ± 402 KO terms, and the metagenomes of HMP mock communities of 6051 ± 85 KO terms where, for example, 263 ± 131 means an average of 263 KO terms and a standard deviation of 131 KO terms. The number of KO terms present in sequenced metagenomes can be affected by sequencing depth. Therefore, the small number of KO terms identified in a sequenced metagenome may lead to erroneous estimates of community functional diversity, which makes it difficult to validate metagenome prediction tools. For the comparison of PanFP–PICRUSt, we used PICRUSt-compatible closed OTU-tables (97 % identity via uclust [21]). PICRUSt makes use of a 16S rRNA gene phylogeny of all OTUs available in a reference database (Greengenes [22]), along with the functional composition of reference genomes assigned to these OTUs (IMG [23]). From this combined information, PICRUSt infers function content at the ancestral nodes based on the 16S rRNA gene phylogeny. Therefore, the PICRUSt-compatible OTU-tables can only be constructed from the closed-reference OTU picking protocol. We note that Nelson et al. [24] reported that the closed-reference OTU picking protocol introduces biases since a large number of reads (on average 35 % with large variance across different samples) which fail to be assigned to reference OTUs are discarded. Based on a PICRUSt-compatible OTU-sample table, we inferred a functional profile for each sample. Note that it took much less than one minute for functional profiles to be generated via PanFP for the HMP mock community samples on a laptop 2.6 GHz CPU. Figure 1 shows the Spearman rank correlation between our inferred functional profiles and sequenced metagenomic functional profiles. Note that we considered only KO terms with non-zero estimates in both functional profiles being compared when calculating Spearman correlation in the study since functional profiles were represented by different sets of KO terms by different approaches. The Spearman rank correlation between the predicted PanFP and actual metagenome functional profiles improved with the increase in the number of KO terms identified in sequenced metagenomes, which corresponds to an increase of sequencing depth. This can be directly observed in our comparison of the two HMP mock communities, which showed the deepest sequencing depth among the studied samples, with correlations of ≃0.9. For all 65 samples, correlations and the number of KO terms common in both profiles are summarized in Additional file 1: Table S1. As expected, the hypersaline mat samples, which were shallowly sequenced, showed artificially low correlations with our inferred functional profiles.

**Fig. 2:**
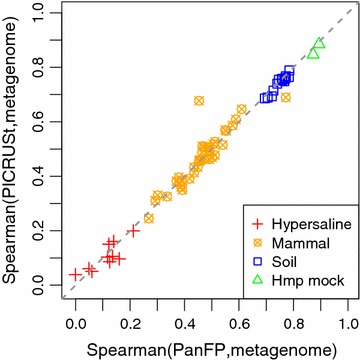
A *scatter plot* of the two Spearman correlations for 65 samples: X-axis and Y-axis represent correlations between functional profiles inferred by PanFP and PICRUSt versus sequenced metagenomic functional profiles, respectively

Also, we compared our inferred functional profiles to those produced from PICRUSt. Figure 2 is a scatter plot of the two Spearman correlations for 65 samples where X-axis represents correlation between functional profiles inferred by PanFP and sequenced metagenomic functional profiles, and Y-axis represents correlations between functional profiles inferred by PICRUSt and sequenced metagenomic functional profiles. Both of metagenome prediction methods essentially show identical performance (with *p* value of 0.98 by Welch two sample *t* test) for almost all samples. PanFP and PICRUSt build very similar functional profiles whose Spearman rank correlations considering KO terms common to both profiles are shown in Additional file 1: Figure S3 where correlations have an average of 0.85. A sample labeled as Squirrel from mammal showed a much higher correlation with PICRUSt than PanFP. The two KO terms with the highest abundance in the squirrel metagenome were K07024 (whose definition is not described) and K02029 (whose definition is polar amino acid transport system permease protein). The abundance of those two KO terms showed better agreement with inferred functional profile by PICRUSt than by PanFP. For samples from HMP mock communities and soil which have better sequencing depth in terms of the number of KO terms identified compare to other test samples in our study, PanFP showed slightly better correlation with metagenomic functional profiles than PICRUSt.

### Scripts

PanFP includes a database of prokaryotes genomes with functional annotation by KEGG Orthology, and several Perl scripts. We provide a single easy to use script, which implements the entire PanFP pipeline from start to finish, as wells as several intermediate scripts for advanced users, so that they may tailor PanFP to their needs. For a given OTU profile, scripts perform each step with a negligible computational time as described in the “Algorithm details” section.

## Conclusions

We presented a new pipeline called PanFP for inferring the functional composition of microbial communities from 16S rRNA gene survey data. These inferred profiles are based on the construction of a pangenome through the OTUs’ taxonomic lineages PanFP can be extended to other phylogenetic marker genes in a similar manner. While PanFP and PICRUSt share many similarities in approach and results, there are several differences: (1) PanFP is not limited to a specific OTU-picking protocol, (2) PanFP is not confined to specific reference genomes/complete genomes, and (3) PanFP should be less sensitive to horizontal gene transfer within an OTU’s pangenome. The accuracy of our method is primarily affected by external factors, such as the taxonomic resolution of OTUs, and the availability and diversity of microbial organisms with robust functional annotation. PanFP will become increasingly more robust as the depth and breadth of microbial databases continue to grow. Note that these issues would also apply to the performance of any other metagenome prediction tools based on 16S rRNA community data.

PanFP showed a remarkable accuracy for HMP mock communities with deeply sequenced metagenomes such that PanFP would provide a cost-effective new way to study complex ecosystems through comparative functional metagenomics and metadata analysis. Within the limitations described above, these comparisons should provide a statistically robust framework for highlighting the most over or under represented biological functions. Also, PanFP can serve as a first step in large metagenomics projects to identify potential samples of interest for direct metagenome sequencing.

## Availability and requirements

- Project name: PanFP
- Project home page: https://github.com/srjun/PanFP
- Operating systems(s): Linux, Mac OS X
- Programming language: Perl
- License: GNU
- Any restrictions to use by non-academics: None.

## Availability of supporting data

The data sets supporting the results of this article are included within the article and its additional files.

## Additional file

10.1186/s13104-015-1462-8 This is a doc file which includes all supporting results (Figure S1, Figure S2, Figure S3, and Table S1).

## Abbreviations

OTU: operational taxonomic unit
KO: KEGG Orthology
UniProtKB: UniProt KnowledgeBase
NCBI: National Center for Biotechnology Information
PanFP: pangenome-based functional profile
