# Supplementary material for: PanFP: pangenome-based functional profiles for microbial communities
Source: BMC Res Notes. 2015 Sep 26;8:479. doi: 10.1186/s13104-015-1462-8 (PMC4584126; doi:10.1186/s13104-015-1462-8)
Supplement: Supplementary file 1 — 10.1186/s13104-015-1462-8 This is a doc file which includes all supporting results (Figure S1, Figure S2, Figure S3, and Table S1). [file 13104_2015_1462_MOESM1_ESM.docx]

**PanFP: Pangenome-based functional profiles for microbial communities**

Se-Ran Jun, Michael S. Robeson, Loren J. Hauser, Christopher W. Schadt, Andrey A. Gorin

**Figure S1.** Flow diagram of PanFP.

**
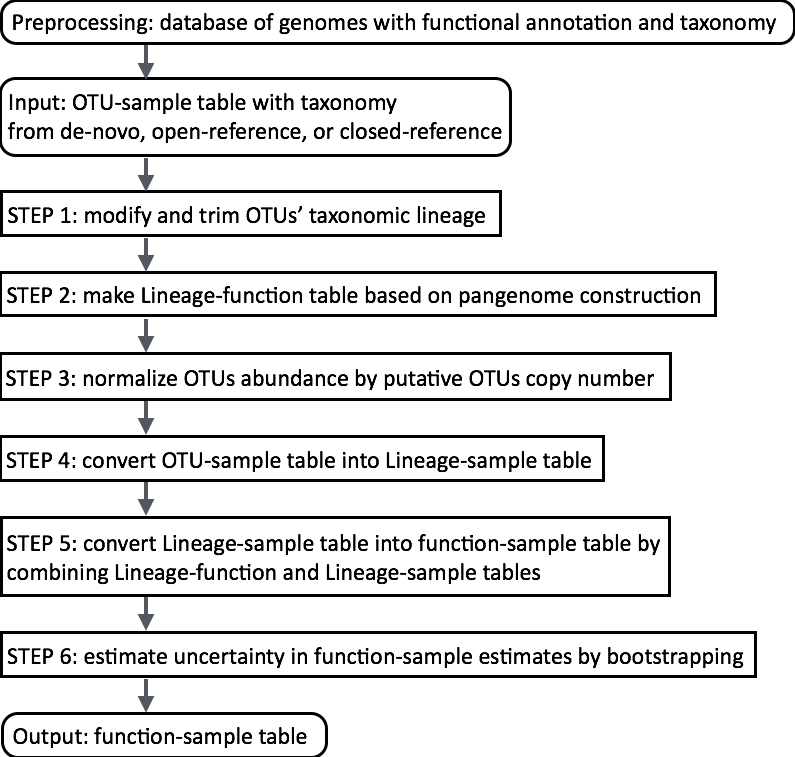
**

**Figure S2. ~~Figure S1~~.** Functional coverage. The X-axis represents the functional coverage, which is defined as the number of proteins with functional annotations by KO terms divided by the total number of proteins for each organism. The Y-axis represents the number of organisms whose complete proteomes have the given functional annotation percentage. The functional profiles of samples by PanFP depend on pangenome construction. Therefore, the occurrence of functional terms is related to the number of genomes included in pangenome construction. But, the number of genomes involved in different pangenomes varies greatly. Therefore, we normalized occurrence of functional terms by the number of genomes pooled for pangenome construction. We filtered out genomes with poor functional annotation to avoid the underestimation of frequency of functional terms, and excluded 252 complete prokaryotes with a 30% cutoff for KEGG Orthology. However, with a 10% cutoff, we excluded 253 complete prokaryotes for KEGG Orthology, indicating that we employed almost all annotated genomes of complete prokaryotes.

**
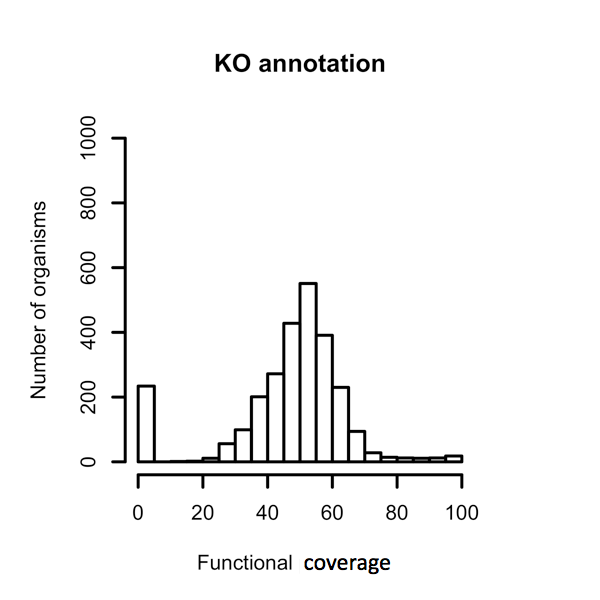
**

**Figure S3. ~~Figure S2~~.** Spearman rank correlation between PanFP and PICRUSt.

**Table S1.** Summary of the number of KO terms with positive estimates in functional profiles. The table summarizes the number of KO terms with positive estimates in functional terms by sequenced metagenome, PanFP, and PICRUSt for 65 samples. ~~Summary of the number of KO terms in functional profiles by sequenced metagenome, PanFP and PICRUSt.~~

| Hypersaline  sampleID | Meta  genome | PanFP | PICRUSt | PanFP  vs  Meta  genome | PICRUSt  vs  Meta  genome | PanFP  vs  PICRUSt | Corr  PanFP  vs  Meta  genome | Corr  PICRUSt  vs  Meta  genome | Corr  PanFP  vs PICRUSt |
| --- | --- | --- | --- | --- | --- | --- | --- | --- | --- |
| 4440968.3 | 167 | 6247 | 3948 | 165 | 162 | 3718 | 0.122 | 0.151 | 0.805 |
| 4440972.3 | 178 | 6888 | 4198 | 177 | 177 | 4000 | 0.160 | 0.097 | 0.806 |
| 4440970.3 | 182 | 6444 | 3952 | 181 | 179 | 3737 | 0.125 | 0.086 | 0.820 |
| 4440971.3 | 190 | 6322 | 3933 | 190 | 187 | 3732 | 0.140 | 0.161 | 0.788 |
| 4440969.3 | 198 | 6305 | 3951 | 197 | 197 | 3746 | 0 | 0.039 | 0.832 |
| 4440964.3 | 556 | 6195 | 3970 | 553 | 549 | 3828 | 0.212 | 0.2 | 0.835 |
| 4440967.3 | 172 | 5614 | 3909 | 171 | 169 | 3679 | 0.135 | 0.106 | 0.823 |
| 4440966.3 | 263 | 6328 | 3926 | 262 | 258 | 3727 | 0.049 | 0.061 | 0.826 |
| 4440965.3 | 301 | 6320 | 3866 | 300 | 289 | 3754 | 0.06 | 0.051 | 0.841 |
| 4440963.3 | 424 | 6606 | 4196 | 421 | 420 | 3997 | 0.117 | 0.103 | 0.84 |
| Soil  sampleID | Meta  genome | PanFP | PICRUSt | PanFP  vs  Meta  genome | PICRUSt  vs  Meta  genome | PanFP  vs  PICRUSt | Corr  PanFP  vs  Meta  genome | Corr  PICRUSt  vs  Meta  genome | Corr  PanFP  vs  PICRUSt |
| SV1 | 4966 | 6054 | 4672 | 4034 | 3835 | 4418 | 0.770 | 0.761 | 0.874 |
| TL1 | 4325 | 6222 | 4438 | 3801 | 3589 | 4253 | 0.783 | 0.764 | 0.885 |
| SF2 | 4497 | 6497 | 4858 | 3892 | 3787 | 4658 | 0.760 | 0.753 | 0.856 |
| BZ1 | 4723 | 6195 | 4593 | 3824 | 3679 | 4347 | 0.772 | 0.767 | 0.883 |
| PE6 | 4873 | 6928 | 5130 | 4367 | 4179 | 4986 | 0.785 | 0.789 | 0.891 |
| CL1 | 4609 | 6463 | 4657 | 3893 | 3757 | 4522 | 0.757 | 0.758 | 0.878 |
| EB026 | 4114 | 5936 | 4441 | 3700 | 3566 | 4283 | 0.766 | 0.749 | 0.841 |
| EB021 | 3476 | 6280 | 3643 | 3170 | 2845 | 3528 | 0.705 | 0.689 | 0.820 |
| AR3 | 4047 | 6517 | 4614 | 3728 | 3519 | 4432 | 0.695 | 0.686 | 0.832 |
| EB020 | 3892 | 6300 | 4327 | 3518 | 3319 | 4140 | 0.721 | 0.694 | 0.813 |
| KP1 | 4183 | 6850 | 4981 | 3846 | 3746 | 4776 | 0.747 | 0.755 | 0.860 |
| EB024 | 4105 | 6379 | 4513 | 3697 | 3498 | 4323 | 0.726 | 0.715 | 0.812 |
| MD3 | 4484 | 6478 | 4890 | 3819 | 3702 | 4685 | 0.768 | 0.757 | 0.869 |
| DF1 | 4293 | 6454 | 4523 | 3710 | 3603 | 4377 | 0.740 | 0.74 | 0.859 |
| Mammal  sampleID | Meta  genome | PanFP | PICRUSt | PanFP  vs  Meta  genome | PICRUSt  vs  Meta  genome | PanFP  Vs  PICRUSt | Corr  PanFP  vs  Meta  genome | Corr  PICRUSt  vs  Meta  genome | Corr  PanFP  vs  PICRUSt |
| Capybara | 1333 | 5737 | 3653 | 1258 | 1260 | 3459 | 0.590 | 0.609 | 0.861 |
| GorillaSTL | 662 | 5736 | 3531 | 637 | 631 | 3447 | 0.370 | 0.382 | 0.871 |
| Hyena2 | 1455 | 5930 | 3683 | 1434 | 1405 | 3595 | 0.549 | 0.57 | 0.854 |
| BaboonSTL | 728 | 5462 | 3274 | 710 | 704 | 3181 | 0.393 | 0.384 | 0.869 |
| Echidna | 1868 | 6138 | 4659 | 1849 | 1845 | 4537 | 0.767 | 0.76 | 0.894 |
| PolarBr2 | 3145 | 5868 | 4350 | 3122 | 3088 | 4250 | 0.502 | 0.469 | 0.873 |
| Saki | 1603 | 6062 | 4055 | 1480 | 1451 | 3972 | 0.576 | 0.587 | 0.853 |
| Orang1 | 747 | 5785 | 4054 | 715 | 702 | 3955 | 0.456 | 0.459 | 0.879 |
| BushDog1 | 1421 | 4334 | 2931 | 1388 | 1362 | 2758 | 0.553 | 0.566 | 0.843 |
| VWPig | 932 | 5204 | 3152 | 916 | 897 | 3017 | 0.454 | 0.458 | 0.860 |
| Okapi1 | 656 | 6004 | 3569 | 646 | 636 | 3472 | 0.382 | 0.377 | 0.841 |
| Horse1 | 976 | 6358 | 4839 | 941 | 944 | 4601 | 0.476 | 0.466 | 0.876 |
| AfElphSD3 | 991 | 5264 | 2926 | 938 | 897 | 2846 | 0.448 | 0.433 | 0.847 |
| BigHornSD | 743 | 5873 | 3562 | 729 | 722 | 3453 | 0.379 | 0.396 | 0.862 |
| ZebraSTL1 | 605 | 6324 | 3337 | 600 | 593 | 3273 | 0.301 | 0.331 | 0.861 |
| Kroo3 | 549 | 5646 | 3453 | 547 | 543 | 3326 | 0.269 | 0.245 | 0.840 |
| Callimicos | 1214 | 4941 | 2968 | 1201 | 1169 | 2864 | 0.512 | 0.478 | 0.813 |
| BaboonW | 609 | 5094 | 2727 | 600 | 588 | 2632 | 0.392 | 0.35 | 0.810 |
| HyraxSD | 986 | 5543 | 3840 | 959 | 947 | 3734 | 0.490 | 0.462 | 0.847 |
| BlackRhino1 | 1050 | 4671 | 2923 | 1024 | 1008 | 2783 | 0.510 | 0.518 | 0.861 |
| Urial2 | 697 | 5650 | 3260 | 669 | 661 | 3156 | 0.418 | 0.391 | 0.847 |
| Chimp2 | 499 | 5384 | 3074 | 482 | 470 | 2975 | 0.336 | 0.326 | 0.853 |
| BlackBr2 | 2975 | 5381 | 3502 | 2942 | 2836 | 3452 | 0.610 | 0.646 | 0.809 |
| SpgbkW | 1238 | 6381 | 3678 | 1219 | 1199 | 3593 | 0.490 | 0.496 | 0.851 |
| SpecBr2 | 1858 | 5136 | 2206 | 1838 | 1386 | 2174 | 0.772 | 0.69 | 0.842 |
| Squirrel | 1361 | 4444 | 2791 | 1348 | 1334 | 2671 | 0.453 | 0.677 | 0.695 |
| Colobus | 954 | 5585 | 3187 | 925 | 905 | 3094 | 0.453 | 0.455 | 0.822 |
| HyraxSTL | 1122 | 5490 | 3243 | 1077 | 1069 | 3162 | 0.472 | 0.507 | 0.853 |
| Gazelle3 | 929 | 5347 | 3110 | 886 | 872 | 3006 | 0.435 | 0.433 | 0.851 |
| BigHornW3 | 1191 | 5702 | 3364 | 1100 | 983 | 3276 | 0.387 | 0.361 | 0.829 |
| Chimp1 | 1192 | 5689 | 3355 | 1106 | 1068 | 3257 | 0.512 | 0.527 | 0.869 |
| Lion2 | 3031 | 5719 | 4015 | 2984 | 2918 | 3915 | 0.462 | 0.46 | 0.825 |
| Rabbit | 912 | 4766 | 2708 | 902 | 886 | 2599 | 0.435 | 0.413 | 0.831 |
| Okapi2 | 554 | 5747 | 3241 | 536 | 533 | 3149 | 0.293 | 0.311 | 0.861 |
| Armadillo | 1066 | 5634 | 4198 | 1056 | 1055 | 4076 | 0.487 | 0.506 | 0.856 |
| Giraffe2 | 905 | 6242 | 3836 | 876 | 862 | 3752 | 0.502 | 0.491 | 0.869 |
| RTLemur | 1136 | 6263 | 3706 | 1125 | 1114 | 3622 | 0.470 | 0.511 | 0.850 |
| Lion1 | 1273 | 5414 | 4069 | 1261 | 1254 | 3943 | 0.464 | 0.488 | 0.830 |
| BlackLemur | 1245 | 4813 | 3024 | 1215 | 1201 | 2880 | 0.540 | 0.515 | 0.872 |
| HMP mock  sampleID | Meta  genome | PanFP | PICRUSt | PanFP  vs  Meta  genome | PICRUSt  vs  Meta  genome | PanFP  Vs  PICRUSt | Corr  PanFP  vs  Meta  genome | Corr  PICRUSt  vs  Meta  genome | Corr  PanFP  vs  PICRUSt |
| even | 6111 | 6550 | 5432 | 5152 | 5020 | 5279 | 0.894 | 0.885 | 0.910 |
| staggered | 5991 | 6678 | 5485 | 5109 | 4984 | 5345 | 0.872 | 0.847 | 0.914 |
